# A Novel Approach in the Development of Larval Largemouth Bass *Micropterus salmoides* Diets Using Largemouth Bass Muscle Hydrolysates as the Protein Source

**DOI:** 10.3390/ani13030373

**Published:** 2023-01-21

**Authors:** Giovanni S. Molinari, Michal Wojno, Genciana Terova, Macdonald Wick, Hayden Riley, Jeffrey T. Caminiti, Karolina Kwasek

**Affiliations:** 1 Center for Fisheries, Aquaculture, and Aquatic Sciences, Southern Illinois University, Carbondale, IL 62901, USA(Michal Wojno); 2 Department of Biotechnology and Life Sciences, University of Insubria, Via Dunant, 3-21100 Varese, Italy; 3 Department of Animal Science, The Ohio State University, Columbus, OH 44691, USA(Macdonald Wick);

**Keywords:** protein hydrolysates, larval nutrition, live feed replacement, largemouth bass, skeletal deformities, free amino acids

## Abstract

**Simple Summary:**

During the larval stage, Largemouth Bass rely heavily on the live feed. However, live feed culture is expensive to maintain, requires more labor, and is highly unpredictable. Dry diets would provide more nutritional control during the larval culture and be more cost-effective than live feed. Hence, live feed replacement has been a major focus of the aquaculture industry in the past decades. Larval Largemouth Bass is characterized by a highly underdeveloped gut, unable to efficiently digest complex dietary protein, and the amino acid requirements of larval fish in general are difficult to quantify. This study proposed the utilization of adult Largemouth Bass muscle as a protein source to provide the optimal amino acid composition for the larval fish of the same species. The muscle was also predigested with enzymes from the adult bass gut to produce protein that would be easier to digest by the primitive larval gut. Overall, the innovative protein did not improve the growth performance of the larval Largemouth Bass. Further research into determining the optimal protein source and its form is recommended to reduce the reliance on live feeds during the Largemouth Bass’ larval stage.

**Abstract:**

This study’s objectives were to determine the effect of Largemouth Bass (LMB) muscle hydrolysates obtained using same-species digestive enzymes and the degree of LMB muscle hydrolysis when included in the first feeds of growth performance and survival, skeletal development, intestinal peptide uptake, and muscle-free amino acid composition of larval LMB. LMB muscle was mixed with digestive enzymes from adult LMB, and hydrolyzed for 1.5, 3, and 6 h, respectively. Five diets were produced, the intact diet containing non-hydrolyzed muscle and four diets with 37% muscle hydrolysate inclusion. Those diets were characterized by their level of each hydrolysate (presented as a ratio of 1.5, 3, and 6 Ts hydrolysates): 1:1:1, 1:3:6, 1:3:1, 6:3:1 for diets A, B, C, and D, respectively. To account for gut development, one group of larval LMB was fed a weekly series of diets B, C, and D to provide an increasing molecular weight profile throughout development. This group was compared against others that received either; (1) diets D, C, and B; (2) diet A; or (3) intact diet. The initial inclusion of the hydrolysates significantly improved the total length of the larval LMB; however, neither the hydrolysate inclusion nor the series of dietary molecular weight profiles improved the overall growth of larval LMB. The inclusion of hydrolysates significantly decreased the occurrence of skeletal deformities. The degree of hydrolysis did not have a significant effect on the parameters measured, except for intestinal peptide uptake, which was increased in the group that received the most hydrolyzed diet at the final time of sampling. The lack of overall growth improvement suggests that while the hydrolysates improve the initial growth performance, further research is necessary to determine the optimal molecular weight profile, hydrolysate inclusion level, and physical properties of feeds for larval LMB.

## 1. Introduction

Currently, the successful larval rearing of many marine and freshwater species relies heavily on the live feed, such as algae, rotifers, copepods, and *Artemia* nauplii. Live feed provides significant advantages, such as prolonged availability in the water column and the ability to trigger the natural feeding response of larvae through quick, jerky movements [1]. However, this heavy reliance on live feed poses major challenges to the aquaculture industry. Live feed is much more labor-intensive and expensive than dry-formulated diets [2,3,4] and the nutritional profiles of live feed are inconsistent and may lack essential nutrients [5,6]. Thus, to alleviate the reliance on the live feed, research has focused on developing optimal formulated diets for larval fish able to sustain high growth rates and survival. 

Typically, replacing live feed with dry diets has resulted in significantly reduced growth and survival in both marine [4,7,8,9,10,11,12] and freshwater species [13,14,15,16]. Additionally, skeletal deformities, including scoliosis and lordosis, have increased significantly in larval fish fed with a dry diet [17]. The hindered performance of larval fish on dry feed has been attributed to two major factors. Firstly, the nutritional requirements of larval fish are difficult to quantify. To estimate the optimal amino acid profile for the larval fish, analysis of the amino acid profile of the adult fish has been suggested [18,19,20]. This concept is based on the “Ideal Protein” [21], which assumes that the optimal amino acid requirements of a fish are reflected by its whole-body amino acid profile [22]. This suggests that the muscle of the adult fish contains the optimal amino acid composition required by its larval stage and, hence, the adult stage muscle fulfills requirements for the exact amino acid profile required for larval growth and development. Secondly, in larval fish, the capacity to digest protein is reduced and the larvae are not able to efficiently break down and absorb intact dietary proteins [23,24]. To combat this, protein hydrolysates have been suggested as an optimal protein source for larval fish [24]. The inclusion of protein hydrolysates in formulated dry diets has been tested previously and positive results on growth performance and the occurrence of skeletal deformities have been observed at dietary inclusion levels lower than 50% [17,25,26,27]. In addition to inclusion level, the protein sources used, conditions (pH, temperature), and the enzymes utilized are all critical in successful protein hydrolysate production and utilization [28]. All of these factors can determine not only the protein profile of the hydrolysate, but also its palatability and proportion of polypeptides, oligopeptides, free amino acids (FAA), etc. [29], which can significantly impact how efficiently larval fish utilize hydrolysate-based diets [28,30]. Kwasek et al. [27] used Bighead carp (*Hypophthalmichthys nobilis*) muscle as a protein source in the diets of larval Largemouth Bass (*Micropterus salmoides*) (LMB). The carp muscle was hydrolyzed with the endogenous enzymes from adult LMB in vitro and its dietary inclusion improved growth performance and reduced skeletal deformities of the larval LMB [27]. However, the live feed could not be fully replaced using the carp hydrolysate-based diet, possibly suggesting that the protein source used for larval LMB diets should more closely match its nutritional requirements. 

Another consideration in developing an optimal formulated dry diet for larval fish is how the digestive tract changes throughout development during the larval stage. As the digestive tract matures, a shift in enzymatic activity occurs from higher levels of peptidases designed to break down smaller peptides, towards increased levels of enzymes, such as pepsin (gastric species) and trypsin (gastric and agastric species), that function to break down intact proteins into smaller peptides [23,24]. Based on this, the optimal protein sizes for larval fish increase as the fish metamorphose and the digestive tract develops [31]. Canada et al. [31] observed that pre-metamorphic Senegalese Sole (*Solea senegalensis*) utilized lower-molecular-weight peptides (5–70 kDa) better than larger, intact proteins. In comparison, post-metamorphic Sole absorbed the intact protein more efficiently than the lower-molecular-weight fractions. Thus, providing larval fish with the right proportion of FAA and/or di-/tripeptides and then shifting to larger polypeptides and or intact protein as metamorphosis continues seems to present an ideal feeding regimen for optimal larval development.

To address these challenges and develop an optimal formulated dry diet and feeding regimen for larval LMB, this study had two objectives; (1) to evaluate the utilization of same-species muscle hydrolyzed with same-species digestive enzymes as a protein source in the diets for larval LMB; and (2) to investigate how the molecular weight profile of the dietary protein affected utilization throughout larval development. These objectives were evaluated based on growth performance and survival, the occurrence of skeletal deformities, the expression of intestinal peptide transporter PepT1, and postprandial muscle FAA composition used as an indicator of dietary amino acid availability. 

## 2. Materials and Methods

### 2.1. Experimental Conditions

The feeding trial was conducted at the Center for Fisheries, Aquaculture, and Aquatic Sciences at Southern Illinois University-Carbondale (SIUC), IL. All experiments were carried out in strict accordance with the recommendations in the Guide for the Care and Use of Laboratory Animals of SIUC. The SIUC Institutional Animal Care and Use approved all of the protocols performed (protocol #18–051). The experiment was carried out using a semi-recirculated aquaculture system with two mechanical (sand) filters (Pentair, Minneapolis, MN, USA) and a bio-filter. The system consisted of 30 (280 L) black tanks. The photoperiod was set to 14 h of darkness and 10 h of light, with the overhead lights on from 8 a.m. to 6 p.m. The system was supplied with groundwater, with a constant inflow of 100 mL/min into each tank. During the study, the temperature was 24.72 °C (±0.79) and the pH was 8.45 (±0.08). The salinity of the system was kept at 1–3 ppt in order to prolong the viability of the live feed [32].

### 2.2. Muscle Hydrolysis

The hydrolysis method for this study was based on the in vitro method described by Kwasek et al. [27] with some modification. Adult LMB (2-year-old males and females) were kept at 25 °C and fed to satiation (one meal). Approximately 2 h after feeding, the fish were euthanized with an excess of tricaine methanesulfonate (MS-222) (#E10521, Sigma-Aldrich, St. Louis, MO, USA) with a dose of 0.4 mg/mL. The head and tail of each fish were removed, and their digestive tracts were dissected and placed on ice. The remaining carcass was ground three times with a meat grinder (General Food Service, Weston, FL, USA), diluted with deionized water (1:2), and homogenized with a PowerGen 1000 (Fisher Scientific, Waltham, MA, USA) tissue homogenizer on high speed for 10 min. The digestive tracts were also diluted with deionized water (1:2), homogenized for 10 min, and the homogenates were centrifuged (1500× *g* for 10 min at 4 °C) to obtain the supernatant and to separate it from the solid mass (including undigested feed, fat, and tissues). The supernatant was then immediately used for muscle hydrolysis. 

The LMB muscle homogenates were moved to containers (12 L), placed in a water bath (25 °C), diluted with deionized water (1:2), and stirred using an overhead stirrer (VWR VOS 16, VWR, Radnor, PA, USA) for the duration of the hydrolysis. These muscle homogenates were mixed with the digestive tract supernatant (3:1 muscle:supernatant). This ratio was utilized to match the amount of protein one LMB digestive tract consumes during one meal when fed at a feeding rate of 1% body weight with a commercial diet. Part of the homogenate and supernatant mixture was not incubated and was brought to 90 °C for 15 min immediately after mixing to stop the enzymatic activity. This sample served as the intact protein source (control). Since the intact muscle was exposed to enzymes for a limited time, it cannot be treated as completely unhydrolyzed tissue. However, it did not undergo the specific controlled hydrolysis process and incubation in different pH conditions. Therefore, it is referred to as intact in this study in order to clearly differentiate it from the muscle that was incubated and underwent the hydrolysis process. The rest of the mixture underwent hydrolysis and was kept at 25 °C for the entire process to match the holding temperature of the bass. The mixture was kept at a pH of 3–4 to mimic stomach digestion and then at a pH of 7–9 for the remainder of the hydrolysis to mimic intestinal digestion. Three different hydrolysates were obtained:

- **1.5 h**: 1 h at pH 3–4, followed by 30 min at pH 7–9;
- **3 h**: 1 h at pH 3–4, followed by 2 h at pH 7–9;
- **6 h**: 1 h at pH 3–4, followed by 5 h at pH 7–9.

Each hydrolysate solution was brought to 90^o^C for 15 min immediately after the hydrolysis process to inactivate the enzymes. The muscle hydrolysates and intact protein were stored at −80 °C and later freeze-dried for subsequent analyses. Both muscle hydrolysates and intact muscle were sent to the Ohio State University for proteomic analyses. 

### 2.3. Diets

Five diets were formulated to contain varying mixtures of different hydrolysates with intact protein. The diets were formulated to be isonitrogenous and isolipidic and meet the essential nutrient requirements of larval fish [33]. The diet formulations are presented in Table 1. Prior to mixing, the dry components of the diet were ground to a fine particle size (~0.25 mm) using a centrifugal mill (Retsch 2 M 100, Haan, Germany). Once the dry components were ground, all ingredients in the diet were mixed (HCM450 Vertical Cutter Mixer, Hobart, Troy, OH, USA) to achieve uniform dispersion. The mixture was extruded (Caleva Extruder 20, Sturminster Newton Dorset, England) to produce “noodles” and the noodles were freeze-dried (Labconco, Kansas City, MO, USA) to remove moisture from the diets. After drying, all pellets were separated by size using a vibratory sieve shaker (Retsch AS 200 Basic, Haan, Germany).

The first diet, the intact diet, did not contain any of the LMB muscle hydrolysates and was solely based on LMB intact protein. The dietary protein in the last four diets was based on 50% intact protein and 50% LMB muscle hydrolysates [27]. Diet A contained an equal proportion of all three of the hydrolysates. Diet B contained a 1:3:6 proportion of the 1.5, 3, and 6 h hydrolysates, respectively. This diet was formulated to have the highest proportion of the most hydrolyzed muscle; thus, it contained higher levels of lower-molecular-weight protein products than the other diets. Diet C contained a 1:3:1 proportion of the 1.5, 3, and 6 h hydrolysates, respectively. This diet had a higher proportion of medium-level hydrolysate protein and, therefore, fewer smaller protein fragments than diet B. The last diet, diet D, contained a 6:3:1 proportion of the 1.5, 3, and 6 h hydrolysates, respectively. This diet had a higher proportion of the least hydrolyzed muscle and more of the larger protein fragments than the other hydrolysate-based diets. The dietary amino acid compositions are presented in Table 2. 

### 2.4. Experimental Design

**Figure 1:**
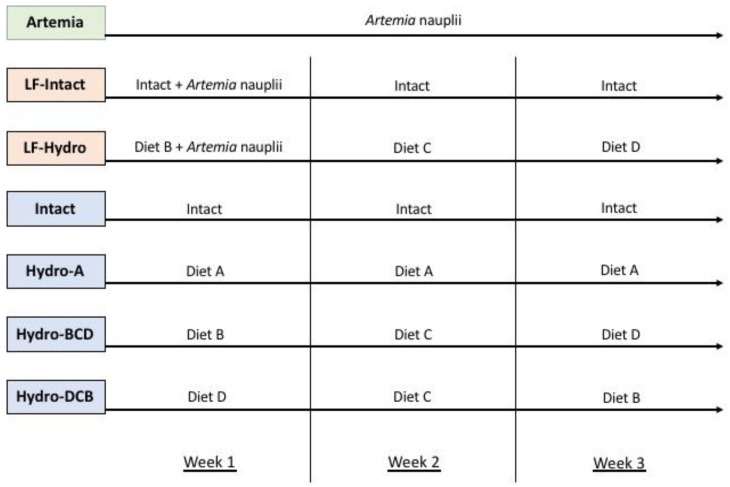
Feeding regimens for experimental groups. Groups in green were fed with live feed, groups in orange were co-fed, and groups in blue were fed dry diets only. All diets were fed in excess during each feeding. The LF-Intact and LF-Hydro groups were fed decreasing amounts of Artemia starting on day 3 to wean them completely onto dry feed starting with week 2. The switch in diets commenced immediately after the sampling and measuring conducted at the end of each week.

At 4 days post-hatch (dph), larval LMB (Little Grassy Fish Hatchery, Makanda, IL, USA) were randomly distributed into 21 (280 L) tanks, with ~10 fish/L. There were seven treatment groups in this study, with three replicate tanks each. Each group was fed to apparent satiation. The feeding regimen for each group is laid out in Figure 1. The experiment was carried out until the fish fully metamorphosed into the juvenile stage (5–26 dph). For the groups with changing diets throughout the study, the diets were switched after weighing the fish at the end of each week. The diets were ground up and fed to the fish in powder form (<250 µm). To ensure constant feed availability during the first week, the larvae were fed every 45 min. After that, during the second week, fish were fed every hour, and during the third week, every hour and a half. Due to high viscosity, the dry feed was added to the surface of the tanks through a sieve (250 µm) to avoid the formation of clumps and ensure proper particle size for consumption. Feed was added to the tank in excess during each feeding. For the tanks that received live feed, *Artemia* was added to the tanks ad libitum and was monitored hourly to ensure a constant supply of food throughout the day. 

The **Hydro-A** group received diet A throughout its development. The next two groups received a series of diets B, C, and D. This is due to the results provided by Canada et al. [31], who found that as larvae develop, their ability to digest and absorb different protein sizes changes. Each of the different hydrolysate-based diets contained varying levels of hydrolyzed protein and, thus, varying molecular sizes of protein. **Hydro-BCD** received a series of hydrolysate-based diets throughout larval metamorphosis that started with diet B during week 1, switched to diet C for week 2, and finished with diet D during week 3. This dietary regimen was applied in order to determine whether providing larval fish with protein in a smaller-molecular-weight form and shifting toward larger sizes further into development would improve digestion and utilization of the proteins from the feeds. The next group, **Hydro-DCB**, was the opposite of the previous group; here, we tested how fish would perform when receiving larger protein fractions at first and then smaller protein fractions as their development continued. These groups received dry diets only, without live food supplementation. The **Artemia** group served as a reference and provided a benchmark for larval growth and performance on only live food (**LF**). The final two groups, **LF-Intact** and **LF-Hydro BCD** were meant to represent how co-feeding with live food impacts the dietary utilization of intact and hydrolysate-based diets, respectively. The **Intact** group received a diet based solely on intact protein, serving as a control for performance without the inclusion of LMB muscle hydrolysates.

### 2.5. Sampling and Measuring

The fish in this study were weighed weekly at the end of weeks 1, 2, and 3. In addition to obtaining the weight data, pictures of the larval LMB were taken during each weighing to gather weekly total length data. ImageJ software (Version 1.53t, NIH, Bethesda, MD, USA) was used to assess the length of fish from the pictures. At the conclusion of the study, 100 fish from each tank were assessed for lordosis, scoliosis, tail, and head/jaw deformities. These deformities are commonly seen in larval fish and are often used as an indicator of nutritional deficiencies [17].

Samples for additional analysis were also taken at the conclusion of the study. Three fish from each tank were euthanized with an overdose of MS-222 and stored in RNAlater (#AM7021, Invitrogen by Fisher Scientific, Waltham, WA, USA) for the analysis of PepT1 expression in the gut. Samples for PepT1 gene expression were taken 24 and 2 h after feeding. Additionally, two sets of whole-body samples were taken from each tank, with three fish per sample. The sampled fish were euthanized in liquid nitrogen and stored at −80 °C for further FAA analysis. These sets of samples were taken 24 and 2 h after feeding and represented basal and postprandial FAA levels, respectively. 

### 2.6. Free Amino Acid Analysis 

FAA analysis of fish tissues was performed according to Kwasek et al. [27]. Muscle samples of three fish from each tank were combined and homogenized together with 0.1 mol/L HCl in 1:9 (w/v) and spun at 12,000× *g* (4 °C, 15 min). Supernatants were collected, filtered (Milipore, 10 kDa cutoff at 15,000× *g*, 4 °C, 30 min), and later diluted with 0.1 mol/L HCl (1:19 v/v) containing norvaline and sarcosine (40 μmol/L) as internal standards. Blanks (0.1 mol/L HCl + 40 μmol/L norvaline and sarcosine) and external standards (Sigma acid/neutral and basic AA) were prepared along with the sample preparation. The same concentration of glutamine in 0.1 mol/L HCl as an external standard was prepared and added to the basic AA standard. Free amino acids were quantified using Shimadzu Prominence Nexera—i LC-2040C Plus (Shimadzu, Japan) according to the Shimadzu protocol No. L529 with modifications. Free amino acid concentrations (expressed as μmol/kg wet body weight) were calculated in LabSolutions software version 5.92 (Shimadzu, Japan) using internal and external standards.

### 2.7. Gene Expression

Gene expression was analyzed using real-time polymerase chain reaction (RT-PCR) as described in Terova et al. [34]. Before the transcript quantification of the PepT1 gene in the LMB intestine, PepT1 in LMB was molecularly cloned and sequenced as the sequence was not available in the GenBank database. 

The primers for the amplification of a partial cDNA sequence of the PepT1 gene in LMB were designed based on the sequence of European sea bass (*Dicentrarchus labrax*) (GenBank acc. n° FJ237043.2). The sequences of the primers (351 bp) were: 5′- GATGACTGTGGGGATGTTCC -3′ for the forward primer and 5′- TCCGGCTTTGATTTGATGTCT -3′ for the reverse primer.

For PCR amplification, an aliquot of cDNA from the LMB intestine was amplified using Advantage^®^ 2 Polymerase Mix (#639201, Takara Bio USA, Inc.) and the designed primers. Thirty-five PCR amplification cycles were set using the T100 Thermal Cycler (BioRad, Segrate, Italy). For cloning, PCR thermal cycling conditions were: initial denaturation at 95 °C for 1 min, followed by 35 cycles at 95 °C for 30 s, 55 °C for 30 s, 68 °C for 1 min, and a final elongation step at 69 °C for 1 min followed by 10 min at 70 °C as suggested for T/A cloning. The PCR amplification product was then loaded onto 2% agarose gel with ethidium bromide in TAE (Tris, Acetic Acid, EDTA) 1X buffer to verify the amplicon length. The band of interest was extracted from the gel and purified using the NucleoSpin^®^ Gel, and PCR Clean-up kit (Macherey-Nagel, Germany). Then, the DNA (insert of interest) was directly ligated to the T-tailed plasmid vector pGEM^®^-T Easy (Promega Milan, Italy) and subsequently sequenced in both directions (T7 and SP6). The alignment with Clustal Omega software of partial PepT1 cDNA sequence from LMB with PepT1 from *D. labrax* showed a high degree of similarity (78%) between the two fish species. Hence, the primers designed on the *D. labrax* sequence were found to be suitable for amplifying the PepT1 gene in LMB cDNA samples, too. 

The RT-qPCR was carried out in a final mix volume of 20 µL, containing 3 µL of LMB cDNA (100 ng), 10 µL of iTaq Universal SYBR^®^ Green Supermix (#1725121, Bio-Rad, Milan, Italy), and 500 nM of each primer, using the CFX96 RT-PCR instrument (Bio-Rad, Milan, Italy), following the manufacturer’s instructions. The reaction thermal conditions were as follows: 95 °C for 1 min, then 40 cycles at 95 °C for 10 s, and 60 °C for 30 s. A blank sample containing nuclease-free water instead of the cDNA template was included in each assay as a negative control. Relative expression levels were calculated using the 2−∆∆CT method and *eEF1a1* as housekeeping (HK) gene. With regard to HK, we tested three constitutive genes, α-tubulin, *eEF1a1*, and *β-actin*, to select the most stable one to normalize the relative quantification of PepT1 expression. Primers for the tested HK genes were designed based on the sequences of each gene available in the Genbank database for LMB, whereas primers for the amplification of the LMB PepT1 gene were designed based on the aforementioned partial cDNA sequence obtained by molecular cloning and sequencing. 

The accession numbers of the sequences used to design primers for HK genes were as follows: XM_038724778.1 for *eEF1a1*, MH018566.1 for *α-tubulin*, and MH018565.1 for *β-actin*. Primers were designed following the instructions given in the SYBR Green Supermix kit. The melting temperature was 60 °C. The open-source Primer3 under default settings was used to design the primers. Moreover, for the best qPCR efficiency, amplicon length for each pair of primers was set between 70 and 150 bp. Primers used to amplify target and HK genes are listed in Table 3. 

**Figure 2:**
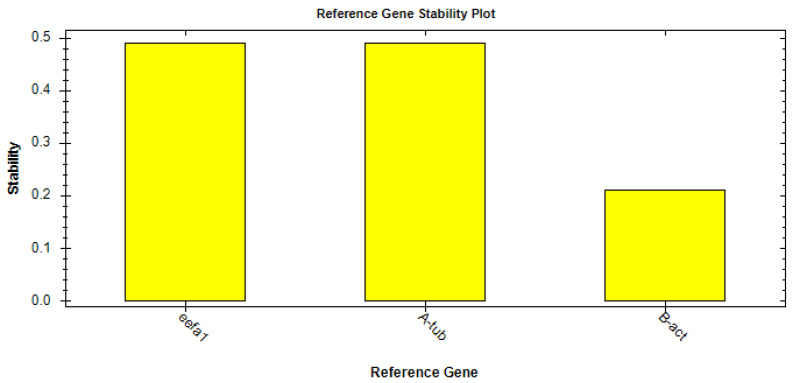
Results of stability analysis of the housekeeping genes eEF1a1, α-tubulin, and β-actin.

By applying the CFX Maestro™ Software (Bio-rad, Milan, Italy), the best reference gene was selected and its stability analyzed by means of the reference gene selection tool (CFX Maestro™ Software User Guide Version 1.1, Bio-rad, Milan, Italy). Following this analysis, the software indicated that *eEF1a1* was the ideal HK based on the average M value (M = 0.48) (Figure 2). 

Amplification reaction efficiency is another important parameter to be considered when performing relative quantitation. Therefore, it was tested for each primer set using, again, CFX Maestro™ Software (Bio-rad, Milan, Italy). Amplification efficiency of a reaction is calculated according to the following equation using data collected from a standard curve: Exponential amplification = 10 (−1/slope)

**Figure 3:**
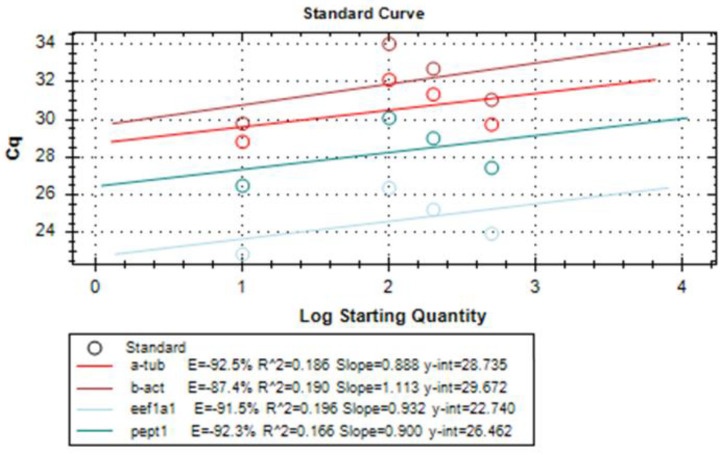
Results of amplification efficiency of each pair of primers for the target and housekeeping genes eEF1a1, α-tubulin, β-actin, and PepT1.

For this, serial dilutions of LMB cDNA were prepared and a standard curve was constructed for each pair of primers. Typically, desired amplification efficiencies range from 90% to 110%. In our case, each primer set showed an amplification efficiency > 80% (Figure 3).

### 2.8. SDS-PAGE

Sodium dodecyl sulfate-polyacrylamide gel electrophoresis (SDS-PAGE) was performed to visualize the products of muscle hydrolysis. Electrophoretic analysis of the samples was conducted on a discontinuous, reducing 12.5% T, SDS-PAGE with modifications of the method described by Updike et al. [35]. Samples were homogenized in dissociation buffer (8 M urea, 2 M thiourea, 60 mM Tris, pH 6.8, containing 3% SDS, 350 mM DDT, and 0.002% bromophenol blue) overnight at 25 °C with agitation and diluted with dissociation buffer as needed. Ten μL of the sample (10 mg/mL) were loaded onto each lane with a 3% stacking gel containing 1% SDS. The proteins were resolved at 150 V cm^−1^ until the dye front reached within 1 mm of the bottom of the gel. The gel was stained with Coomassie Brilliant Blue dye (40% methanol, 5% acetic acid, 0.04% Coomassie Brilliant Blue G-250) overnight and destained with 10% acetic acid. After destaining the gel image was digitized using an AZURE c600 scanner (Azure Biosystems, Dublin, CA, USA). 

### 2.9. Statistical Analysis

The growth performance, survival, and skeletal deformity data collected from this study were analyzed using one-way ANOVA and a Tukey test was used to detect differences between groups. The FAA and PepT1 data were analyzed using a one-way ANOVA and differences between groups were tested using an LSD test. Gene expression variations were analyzed by ANOVA using the PAST3 program (Palaeontological Statistics). In the event of significant differences between the means, the post hoc Tukey or Dunn’s test was applied. The level of significance was set at *p* < 0.05.

## 3. Results

### 3.1. LMB Muscle Hydrolysates 

**Figure 4:**
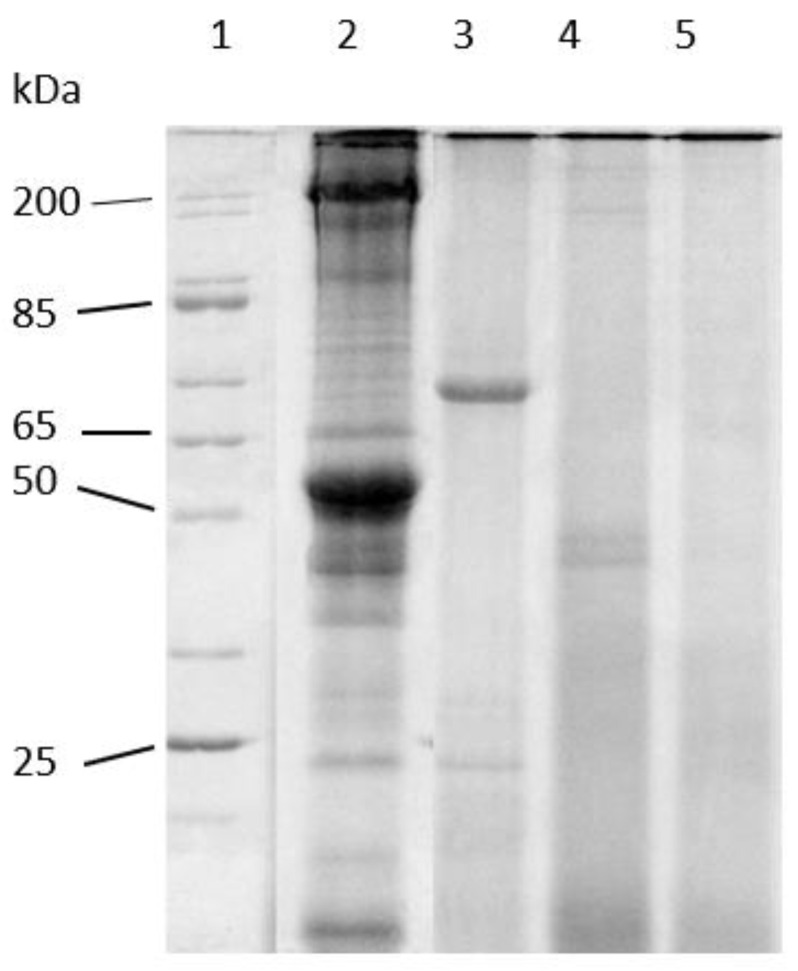
Representative reducing 12.5% T SDS-PAGE of Largemouth Bass muscle hydrolysis over time. Lanes (1) broad range molecular wgt standard (200–10 kDa); (2) intact fish muscle; (3) 1.5 h hydrolysis; (4) 3 h hydrolysis; (5) 6 h hydrolysis.

Figure 4 shows the results of the LMB muscle hydrolysates obtained using endogenous digestives enzymes from adult LMB on a 12.5% SDS-PAGE gel. This concept of hydrolysis has been demonstrated prior by Kwasek et al. [27]. This electrophoretic analysis was performed to confirm the products used in this study were hydrolyzed and to observe any differences that the incubation time had on the hydrolysates. The results from this gel show that the starting material was intact prior to digestion (Lane 2). This demonstrates that without incubation, very little hydrolysis occurred in the time period of harvesting, mixing with the enzymes, and increasing the temperature to 95 °C (heat shock). The intact LMB muscle was incubated with enzymes and samples removed at 1.5 h (Lane 3), 3 h (Lane 4), and 6 h (Lane 5). The hydrolysates were analyzed to observe the degree of hydrolysis over time. Based on these data, the supernatant shows a presence of proteins smaller than 65 kDa initially at 1.5 h. Peptides less than ~45 kDa were remaining after incubation of 3 h and by 6 h it appears that hydrolysis was complete with no observable peptides remaining. This suggests that the proteins had been reduced to small peptides of lower than ~25 kDa. Additionally, this gel confirms that the in vitro muscle hydrolysis process was able to hydrolyze the LMB muscle used in this study. 

### 3.2. Growth and Survival

**Figure 5:**
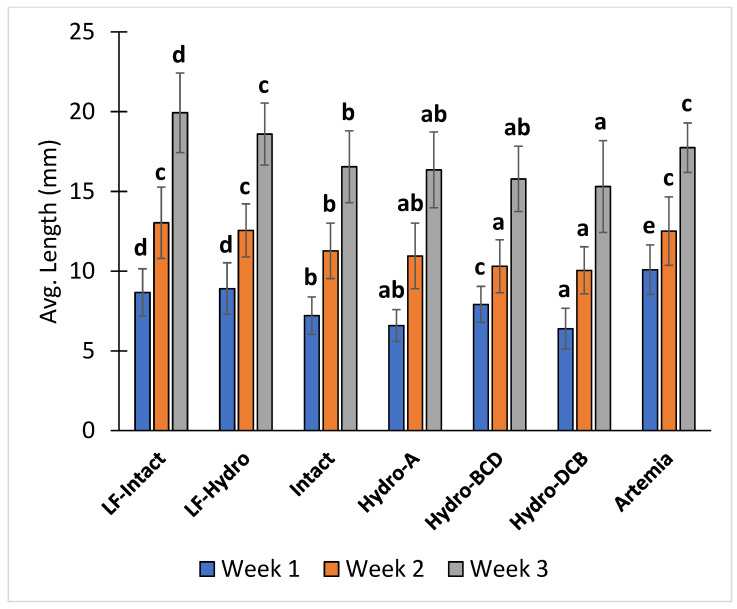
Diet effect on the average total length at the end of each week. Values are presented as means and error bars represent standard deviation for total length. Letters indicate statistical significance between groups. The significance was determined using a One-Way ANOVA and a Tukey test with a *p* value < 0.05.

After the first week of the study (12 dph), the Artemia group had a significantly higher average total length than all other groups. The two LF groups had a significantly higher average total length than the four dry feed-only groups. Among the dry feed-only groups, the Hydro-BCD group had a significantly higher average total length than the other groups, and the Intact group had a significantly higher average total length than the Hydro-DCB group. After the second week (19 dph), the average total lengths of the Artemia, LF-Hydro, and LF-Intact groups were significantly higher than all other groups. Additionally, the Intact group had a significantly higher average total length than the Hydro-BCD and Hydro-DCB groups. At the conclusion of the study (26 dph), the average total length of the LF-Intact group was significantly higher than all other groups, and the LF-Hydro and Artemia groups had significantly higher average total lengths than the four dry feed-only groups. Within the dry feed-only groups, the Intact group had a significantly higher average total length than the Hydro-DCB group. The average total lengths of the Hydro-A and Hydro-BCD groups were not significantly different from either the Intact or Hydro-DCB groups. The average total length results for each week are presented in Figure 5.

At the conclusion of the study (26 dph), the LF-Intact group had a significantly higher average weight than the Hydro-A, Hydro BCD, and Hydro-DCB groups. However, there were no significant differences in final weight among the LF-Hydro, LF-Intact, and Artemia groups. The Artemia group had a significantly higher survival than all other groups, while the LF-Intact group had a significantly higher survival than the LF-Hydro group and the four dry feed-only groups. The LF-Hydro group had numerically higher survival than the Intact group (~10% increase); however, the difference was not significant. The LF-Hydro and Intact groups had significantly higher survivals than the three hydrolysate-based dry feed-only groups. The results for growth performance and survival are presented in Table 4.

### 3.3. Skeletal Deformities 

**Figure 6:**
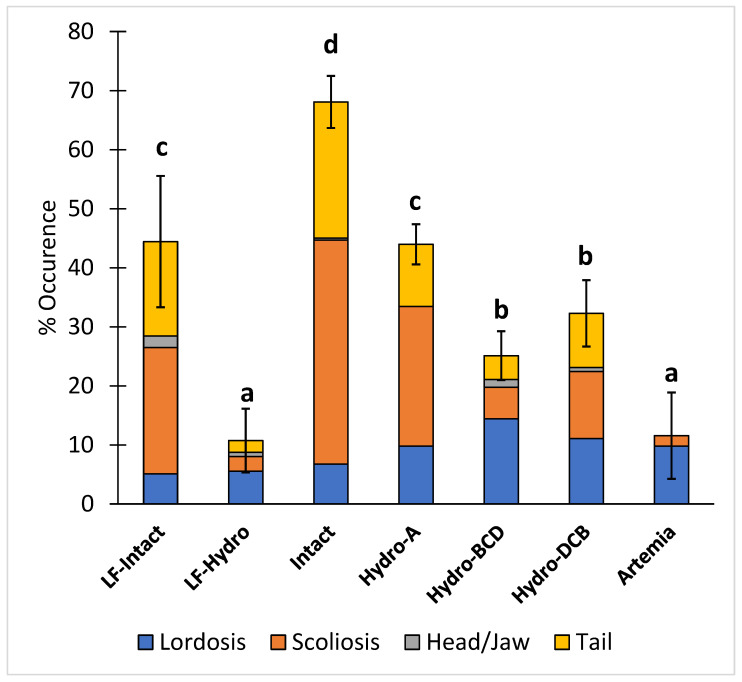
Diet effect on the occurrence of skeletal deformities (Lordosis, scoliosis, and head/jaw and tail deformities). Values are presented as means and error bars represent standard deviation for total deformities. Letters indicate statistical significance between groups. The significance was determined using a One-Way ANOVA and a Tukey test with a *p* value < 0.05.

The occurrence of total skeletal deformities (scoliosis, lordosis, head/jaw, and tail) in the intact group was significantly higher than in all other groups, while there were significantly more deformities in the LF-Intact and Hydro-A groups than in the remaining groups. The LF-Hydro group had a significantly lower occurrence of skeletal deformities compared to all other groups except the Artemia group. There was no significant difference in skeletal deformities between the Hydro-BCD and Hydro-DCB groups. The results for skeletal deformities are presented in Figure 6. 

### 3.4. PepT1 Expression

**Figure 7:**
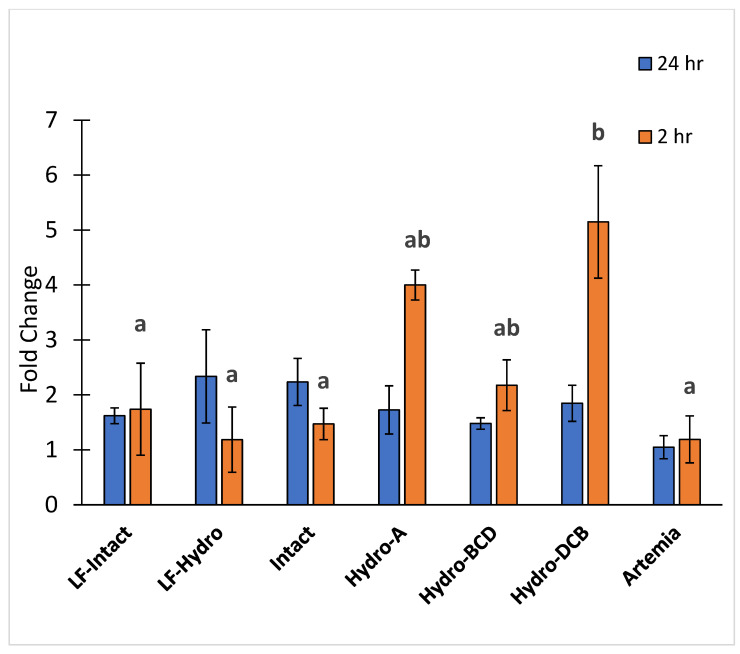
Relative expression of peptide transporter PepT1. Letters indicate statistical significance between groups. The significance was determined using a One-way ANOVA and an LSD test with a *p* value < 0.05.

There were no significant differences in PepT1 gene expression between any of the groups 24 h after feeding. However, gene expression of PepT1 was significantly higher in the Hydro-DCB group compared to every group except Hydro-A and Hydro-BCD 2 h after feeding. The results for PepT1 expression are presented in Figure 7. 

### 3.5. Free Amino Acid Composition

**Figure 8:**
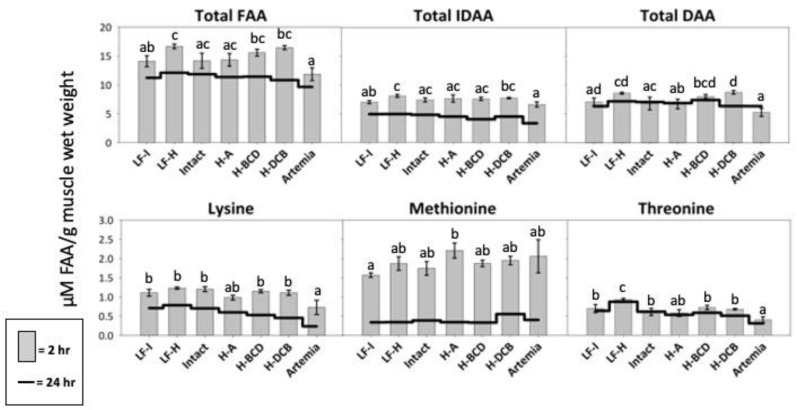
Muscle FAA levels in LMB after 3 weeks of feeding. The gray bars represent FAA levels 2 h after feeding. The black line indicates FAA physiological baseline. Different letters indicate statistical difference at *p* value < 0.05.

The LF-Hydro group presented with significantly higher postprandial levels of total FAA and IDAA in the muscle than the Artemia and LF-Intact groups (Figure 8). The Hydro-DCB group had significantly higher levels of total DAA than the Artemia, Intact, and Hydro-A groups. All groups that received dry feed, except Hydro-A, had a significantly higher level of free lysine than the Artemia group. Significantly higher levels of free threonine were measured in the muscle of the LF-Hydro group than in all other groups, and all groups that received dry feed, except Hydro-A, had a significantly higher level of free threonine than the Artemia group. The Hydro-A group had higher levels of free methionine than the LF-Intact group but did not differ significantly from those in the remaining groups. The Hydro-DCB group showed significantly higher levels of free aspartic acid compared to the Artemia group, and a significantly higher level of free asparagine than the LF-Intact group. The LF-Hydro and LF-Intact groups had significantly higher levels of free glutamic acid compared to the Hydro-A group, while the LF-Hydro group also showed levels significantly higher than those in the Artemia group. The Hydro-BCD and Hydro-DCB had significantly higher levels of free serine than all other groups, except the LF-Hydro group. The Intact group had a significantly higher level of free glutamine than the Hydro-A group. The Artemia group had a significantly lower level of free histidine, tyrosine, arginine, and phenylalanine than all other groups. The LF-Hydro group had a significantly higher level of free glycine than all groups, except the LF-Intact group. The LF-Hydro group also had significantly higher levels of both free arginine and tyrosine than all other groups, except the Hydro-BCD group. The postprandial level of free alanine in the muscle was highest in the Hydro-DCB group, significantly higher than all groups except LF-Hydro and Hydro-BCD. The Hydro-BCD group had the highest level of free phenylalanine, significantly higher than all other groups except for the Intact group. The Hydro-BCD group also had the highest level of free tryptophan, significantly higher than the LF-Hydro, Hydro-A, and Artemia groups. The postprandial level of free proline in the muscle was highest in the Hydro-DCB group, significantly higher than all other groups except for Hydro-A. No significant differences were observed in the postprandial levels of free leucine, isoleucine, valine, and cysteine between groups. The postprandial results for all FAA analyzed are presented in Table 5 (2 h) and Table 6 (24 h).

## 4. Discussion

Kwasek et al. [27] developed an in vitro hydrolysis method utilizing endogenous digestive enzymes from adult LMB. The study found that dietary inclusion of Bighead carp muscle hydrolyzed with the LMB endogenous enzymes significantly improved the growth of larval LMB at a 37% inclusion level [27]. The present study utilized the same in vitro hydrolysis method using LMB endogenous digestive enzymes, and instead of hydrolyzing carp muscle, hydrolyzed the muscle from adult LMB to provide the larval LMB with an optimal dietary amino acid profile. Additionally, the present study utilized LMB muscle that underwent varying degrees of hydrolysis, to provide a wider range of molecular weight of protein products in the diets that would account for the dynamics associated with larval LMB digestive tract development. In contrast to Kwasek et al. [27], the results of the present study did not show a significant improvement in overall growth or survival by including the hydrolysate. The survival results observed in this study are comparable to other studies that utilized protein hydrolysates. Sheng et al. [36] obtained survival levels between 29–43% in larval LMB that were co-fed with live feed during the first five days of the trial, compared to the 33.78% and 46.29% survival levels observed in the co-fed groups (LF-Hydro and LF-Intact) in the present trial. The survival levels observed in the four groups fed dry diets only were markedly lower, ranging from 9–24%. While this survival is very low, it provides an improvement over previous studies that fully replaced live feed with formulated dry diets in Senegalese sole [37] and Red sea bream (*Pagrus major*) [11], both of which experienced no survival within 15 days of feeding with dry diets only. Interestingly, the survival in the hydrolysate-based groups was significantly lower than in the corresponding intact-based groups (i.e., LF-Intact vs. LF-Hydro, and Intact compared to the three hydrolysate-based dry feed groups). This reduction in survival could be attributed to physical differences between the feeds. One major hindrance to weaning larvae onto dry feed is the shortened availability of feed pellets in the water column and lack of predatory response trigger, reducing feed intake [1,38]. Although all the diets were processed in the exact same way, it was observed that the intact diet seemed to disperse more evenly at the surface and sink slower through the water column than the hydrolysate-based diets. This difference in the behavior between the feeds may have increased the feed intake for the groups fed with the intact diet, which could have contributed to the increased survival in the intact groups as compared to the hydro groups. Additionally, the solubility of proteins has been found to rise with increasing degrees of hydrolysis, leading to increased nutrient leaching in the water [39,40,41]. This leaching lessens the nutritional value of the diet and reduces the levels of dietary protein available to the larval fish [41]. Although nutrient leaching from diets to some extent was expected and was accounted for through smaller and more frequent feedings, it is possible that more leaching occurred from the hydrolysate-based feeds, compromising the amino acid composition of the diet and inducing higher mortality in the groups fed with those diets due to potential nutritional deficiency. 

Another possible and more convincing explanation for the difference in results is that Kwasek et al. [27] used only a 3 h hydrolysate in their diets, while this study used a mix of 1.5-, 3-, and 6 h hydrolysates. The composition of protein fragments has a significant impact on the growth of larval fish [28,30,42]. De Vareilles et al. [42] found that a 15% inclusion of larger polypeptides along with 5% of smaller protein sizes (di-/tripeptides, FAA) increased the growth of larval white seabream (*Diplodus sargus*), while 15% inclusion of the small protein fragments and 5% of the large peptides reduced the growth of the larval seabream. The mix of hydrolysates in this study may have been over-hydrolyzed and provided levels of small peptides and FAAs in the diet that were too high, ultimately reducing protein absorption and utilization. This reduction in dietary protein uptake has been attributed to two major factors, an oversaturation of the intestinal protein transporters [43,44,45] and the asynchronous absorption of dietary amino acids [43,45]. The former increases the excretion of amino acids due to their inefficient uptake and the latter reduces protein synthesis due to an imbalanced FAA pool [39,43]. Additionally, Kwasek et al. [27] utilized Bighead Carp muscle as a protein source instead of LMB muscle, which could have also led to differing results. It has been shown that the protein source of hydrolysates used in larval diets significantly affects survival rate [30]. These differences highlight how important it is to determine not only the optimal muscle source, but also the optimal composition of proteins with varying molecular weights when formulating diets for larval fish. While intact protein has been found to be more difficult to digest and utilize by larval fish, compared to hydrolyzed protein, there is a point where a hydrolysate can be over-hydrolyzed and becomes less efficient than the intact protein.

Although we did not observe positive results on overall growth performance and survival in our study, a significant reduction in skeletal deformities occurred with the inclusion of the protein hydrolysate. These deformities, such as scoliosis and lordosis, have been associated with vitamin [26,46], phospholipid [47], and amino acid deficiencies [48]. A high rate of skeletal deformities has a negative impact on production levels at hatcheries, as the deformed fish experience significantly stunted growth and, in more extreme cases, the fish die [49]. In a strictly industrial sense, successful larval rearing would be defined as producing larger and healthier juveniles and adults that can be sold for human consumption or stocking. Fish that have significant deformities are typically disposed of or sold at significantly reduced prices, leading to economic losses for the farmers [26]. The reduction in deformities observed in this study is in agreement with previous studies that investigated hydrolysate inclusion in the diets of larval fish [25,27,50,51,52]. Cahu et al. [50] found that 11% fewer skeletal deformities developed in sea bass larvae with a 58% inclusion of a fish protein hydrolysate in the feed, compared to a diet based solely on intact protein. As observed in this study, the reduction in skeletal deformities does not always result in improved growth performance and survival. Although the LF-Hydro group had a significantly lower level of skeletal deformities, it also had significantly lower weight and survival at the conclusion of the study, compared to the LF-Intact group. Additionally, within the four dry feed-only groups, the Intact group had a significantly higher occurrence of skeletal deformities than the three groups that received the hydrolysate-based diets but had twice the survival. Similarly, Cahu et al. [50] found that the occurrence of skeletal deformities was reduced in larval sea bass with increasing inclusion levels of hydrolysates from 19% to 38% to 58%. However, the inclusion levels of 38% and 58% resulted in significant reductions in survival and growth in the sea bass [50]. This would signify that there seems to be a trade-off between promoting positive growth response and survival versus supporting optimal skeletal development when it comes to the right inclusion level of protein hydrolysates. For example, the LF-Intact group produced the longest and heaviest fish, with 46.29% survival, but 44.23% of the surviving fish exhibited skeletal deformities. Compare that group to the LF-Hydro which experienced a ~12% reduction in weight, 13% lower survival, but a ~33% decrease in skeletal deformities. 

One previously mentioned cause of the reduced growth associated with high levels of low molecular weight protein in the diet is a rapid and asynchronous absorption of dietary amino acids into the body [43,45]. The absorption of dietary amino acids was analyzed in this study by measuring the postprandial FAA composition in the muscle. Previous studies have utilized this parameter as a measure of the availability of dietary amino acids [53], the quality of dietary protein [54,55], and the impact of the molecular form of dietary protein [44]. In this study, an interesting result was the increase in total FAA and IDAA in the muscle 2 h after feeding in the LF-Hydro group, compared to the LF-Intact and Artemia groups. The postprandial increase in FAA in the LF-Hydro group would seem to signify a higher level of absorption of the dietary amino acids from the hydrolysate-based diet compared to the intact-based diet. However, this increase in TFAA and IDAA did not result in an increase in growth compared to those two groups, and thus did not result in increased protein synthesis. This could be due to the asynchronous absorption of the dietary AA, specifically among the IDAA. According to Kwasek et al. [27], the appearance of FAA in the muscle of larval LMB fed with a hydrolysate-based diet seemed to peak earlier than the 3 h postprandial sampling time, compared to the LMB fed with an intact-based diet. Additionally, the authors reported that the FAA in the group fed the intact-based diet peaked around the 3 h sampling time, which would be after the 2 h postprandial sampling time in this study. This is supported by the findings in this study, which show significantly higher levels of TFAA and IDAA at 2 h after feeding in the LF-Hydro group, compared to the LF-Intact group. Since it can be assumed that the muscle FAA has peaked in the LF-Hydro group, one possible explanation for the observed decrease in growth is a delayed peak in threonine compared to the other IDAA. At 2 h after feeding, the threonine level in the LF-Hydro group is only slightly higher than the basal level, while the other IDAA have shown substantial postprandial increases, compared to the basal level. Threonine is one of the three major limiting amino acids in fish, meaning amino acids within the muscle can only be utilized for protein synthesis up to the available level of threonine. This possibly suggests that although the LF-Hydro group had significantly higher postprandial levels of TFAA and total IDAA, the delayed availability of threonine might have been one of the factors that limited protein synthesis and increased protein catabolism. 

Part of the novel approach of this study was also utilizing a series of diets that presented the larval LMB with increasing dietary protein sizes throughout development. This was based on Canada et al. [31] who found that dietary protein size requirements in larval Senegalese sole shift from partially hydrolyzed protein (5–70 kDa) before and during metamorphosis to intact protein after metamorphosis. This was reflected by significantly improved growth in the Senegalese sole, during the metamorphic stage, fed with a diet based on the partially hydrolyzed protein, compared to those fed with the intact protein [31]. After the metamorphic stage, the diet based on intact protein significantly improved the growth of the fish [31]. Based on this outcome, it was suggested that dietary protein should be optimally transitioned from smaller to larger protein fractions over the course of development during the larval stage. This theory was tested in the present study with the Hydro-BCD group, which received a series of diets formulated with increasing protein sizes throughout the feeding trial. The overall results from this group showed that the series of diets provided did not improve the growth or survival of the larval LMB. However, the weekly growth results obtained from this study do support the findings in Canada et al. [31]. After the first week of feeding, the Hydro-BCD group had a significantly higher average total length than the other dry feed-only groups. This suggests that Diet B, which was formulated to have the highest composition of lower molecular weight proteins, was the most efficient diet during the first week of feeding for larval LMB. After the second week of feeding, however, the Hydro-BCD group had a significantly lower average total length than the Intact group. The significant increase in growth of the Intact group during the second week might suggest that at this point in the development of larval LMB, the fish were better suited to feed on diets with larger protein fragments, and the molecular weight profile of Diet C was too low to support optimal growth for the Hydro-BCD group. Previous studies have found that the inclusion of hydrolysates in larval diets stimulated the maturation of the gut and the activity of enzymes associated with protein digestion [28,50]. This maturation of the gut is characterized by a reduction in peptidases and an increase in the activity of enzymes, such as pepsin and trypsin, that break down intact proteins into smaller protein fragments for absorption [23,24]. Based on this, it seems that the initial use of hydrolysates does improve the growth and development of larval LMB; however, after a certain point in the gut maturation, the continued use of hydrolysates becomes inefficient for protein utilization. This is supported by the significant difference in the utilization of Diet B at different points of larval development. Diet B significantly increased the growth performance of the larval LMB during the first week (Hydro-BCD vs. Intact); however, the use of Diet B during the final week of the study significantly decreased the growth of the LMB (Hydro-DCB vs. Intact) and was less efficient than the intact diet. This highlights the importance of providing larger dietary proteins as the LMB progress through larval development. 

While we observed that LMB did utilize increasing protein fragments more efficiently throughout development, the series of diets that were intended to provide the LMB with increasing molecular weight proteins were unable to improve the growth performance over the group that received solely the intact diet. As previously mentioned, we assume that the hydrolysates utilized in this study were over-hydrolyzed and provided dietary protein molecular weight profiles that were much smaller than intended. Canada et al. [31] found that their diet with a high level of low-molecular-weight peptides (<5 kDa) significantly reduced the growth of the larval Senegalese sole compared to the group that received the partially hydrolyzed protein. Previous studies suggest that high inclusion levels of small peptides and FAA lead to an oversaturation of intestinal transporters, reduced protein absorption, and, ultimately, reduced growth [28,31]. It is feasible that both the 3 and 6 h hydrolysates were over-hydrolyzed and provided the diets with too high of a level of low-molecular-weight proteins. This would suggest that diets with high inclusion levels of those two hydrolysates (Diets A, B, and C), although formulated to be different, had molecular weight profiles that were lower than intended and utilized similarly for growth. This theory helps explain the lack of significant differences observed in growth and survival between the Hydro-BCD, Hydro-DCB, and Hydro-A groups. One result observed that signifies the similarities in molecular weight profiles of Diet A and Diet B might be the increase in postprandial expression of PepT1. PepT1 results from Diet C were not obtained as it was not being fed to any of the groups at the conclusion of the study when PepT1 samples were collected. The Hydro-DCB group had a significantly higher expression of PepT1 2 h after feeding compared to all other groups except the Hydro-A and Hydro-BCD groups. Additionally, the Hydro-A group had a numerically higher postprandial expression of PepT1 compared to all other groups, except Hydro-DCB. These results might indicate an increase in di-/tripeptide absorption from diets A and B, the two diets with the highest inclusion levels of the 3 and 6 h hydrolysates. However, the increase in peptide absorption was not reflected in improved growth performance in this study. 

## 5. Conclusions

Overall, the results from this study show that the inclusion of the same-species muscle hydrolysate improved the initial growth of larval LMB but did not improve the overall growth throughout the entire larval stage. It seems that the hydrolysates utilized in this study were over-hydrolyzed and were unable to support optimal growth and survival in larval LMB throughout the fish metamorphosis. To improve the use of same-species muscle hydrolysates as a protein source for larval fish, further research should look into optimizing the hydrolysis process to produce a more ideal molecular weight profile and improve the physical qualities of the diet to ensure high nutrient retention. 

## Figures and Tables

**Table 1:** Diet formulations for feeding trial.

Ingredients (%)	Intact	A	B	C	D
Fish meal (Intact)	74.00	37.00	37.00	37.00	37.00
Hydrolysate 1.5 h	-	12.30	3.70	7.40	22.20
Hydrolysate 3 h	-	12.30	11.10	22.20	11.10
Hydrolysate 6 h	-	12.30	22.20	7.40	3.70
CPSP 90 ^1^	5.00	5.00	5.00	5.00	5.00
Krill Meal ^2^	5.00	5.00	5.00	5.00	5.00
Fish oil ^3^	4.00	4.00	4.00	4.00	4.00
Lecithin ^4^	4.00	4.00	4.00	4.00	4.00
Mineral mix ^5^	3.00	3.00	3.00	3.00	3.00
Vitamin mix ^6^	3.00	3.00	3.00	3.00	3.00
CaHPO_4_	1.00	1.00	1.00	1.00	1.00
Taurine	1.00	1.00	1.00	1.00	1.00
Choline chloride	0.10	0.10	0.10	0.10	0.10
Vitamin C ^7^	0.05	0.05	0.05	0.05	0.05
**Proximate Comp. (%)**					
Crude Protein (*N* × 6.25)	60.97 (±0.08)	60.99 (±0.17)	60.96 (±0.20)	61.02 (±0.03)	60.96 (±0.19)
Lipids	18.05 (±0.18)	18.06 (±0.08)	18.00 (±0.10)	17.98 (±0.16)	17.97 (±0.15)
Ash	12.01 (±0.07)	11.99 (±0.17)	12.02 (±0.15)	12.07 (±0.13)	12.06 (±0.11)

^1^ Soluble fish protein hydrolysate (Sopropeche S.A., Boulogne Sur Mer, France). ^2^ Proccesed Euphausia superba (Florida Aqua Farms, Dade City, FL, USA). ^3^ Cod liver oil (MP Biomedicals, Solon, OH, USA). ^4^ Yelkin TS lecithin (Ingredi Co., Baltimore, MD, USA). ^5^ Bernhart-Tomarelli mineral mix with 5ppm selenium in a form of sodium selenite (Dyets, Bethlehem, PA, USA). ^6^ Custom Vitamin Mixture (mg/kg diet) Thiamin HCl, 4.56; Riboflavin, 4.80; Pyridoxine HCl, 6.86; Niacin, 10.90; D-Calcium Pantothenate, 50.56; Folic Acid, 1.26; D-Biotin, 0.16; Vitamin B12 (0.1%), 20.00; Vitamin A Palmitate (500,000 IU/g), 9.66; Vitamin D3 (400,000 IU/g), 8.26; Vitamin E Acetate (500 IU/g), 132.00; Menadione Sodium Bisulfite, 2.36; Inositol, 500 (Dyets, Bethlehem, PA, USA). ^7^ L-Ascorbyl-2-Polyphosphate (Argent Aquaculture, Redmond, WA, USA).

**Table 2:** Amino acid composition of the formulated diets.

Amino Acid Composition (%)	Intact	A	B	C	D
Alanine	3.90 (±0.10)	3.73 (±0.01)	3.72 (±0.10)	3.73 (±0.09)	3.89 (±0.10)
Arginine	3.54 (±0.08)	3.38 (±0.02)	3.41 (±0.08)	3.38 (±0.08)	3.49 (±0.06)
Aspartic Acid	5.69 (±0.11)	5.49 (±0.02)	5.49 (±0.10)	5.53 (±0.17)	5.35 (±0.09)
Cysteine	0.57 (±0.02)	0.54 (±0.02)	0.57 (±0.01)	0.55 (±0.04)	0.50 (±0.04)
Glutamic Acid	8.02 (±0.19)	7.76 (±0.06)	7.64 (±0.20)	7.70 (±0.16)	7.84 (±0.10)
Glycine	4.73 (±0.20)	4.33 (±0.08)	4.48 (±0.15)	4.45 (±0.15)	4.70 (±0.18)
Histidine	1.35 (±0.02)	1.35 (±0.02)	1.32 (±0.02)	1.32 (±0.05)	1.26 (0.05)
Hydroxyproline	0.87 (±0.07)	0.74 (±0.05)	0.83 (±0.04)	0.79 (±0.18)	1.13 (±0.20)
Isoleucine	2.65 (±0.05)	2.62 (±0.03)	2.56 (±0.05)	2.55 (±0.11)	2.43 (±0.10)
Leucine	4.29 (±0.09)	4.32 (±0.09)	4.15 (±0.08)	4.18 (±0.15)	3.99 (±0.17)
Lysine	4.81 (±0.10)	4.85 (±0.11)	4.65 (±0.09)	4.65 (±0.18)	4.45 (±0.20)
Methionine	1.57 (±0.04)	1.51 (±0.01)	1.49 (±0.04)	1.52 (±0.05)	1.47 (±0.02)
Phenylalanine	2.27 (±0.04)	2.26 (±0.04)	2.19 (±0.04)	2.21 (±0.05)	2.13 (±0.02)
Proline	2.71 (±0.14)	2.42 (±0.07)	2.55 (±0.09)	2.54 (±0.17)	2.89 (±0.24)
Serine	2.12 (±0.04)	2.04 (±0.03)	2.07 (±0.03)	2.10 (±0.04)	2.05 (±0.01)
Taurine	1.95 (±0.27)	2.50 (±0.17)	2.19 (±0.15)	2.23 (±0.14)	2.06 (±0.23)
Threonine	2.49 (±0.05)	2.40 (±0.03)	2.43 (±0.03)	2.46 (±0.07)	2.36 (±0.03)
Tryptophan	0.62 (±0.08)	0.54 (±0.05)	0.45 (±0.08)	0.52 (±0.08)	0.46 (±0.05)
Tyrosine	1.79 (±0.06)	1.86 (±0.06)	1.73 (±0.03)	1.77 (±0.06)	1.68 (±0.09)
Valine	2.98 (±0.05)	2.94 (±0.03)	2.88 (±0.05)	2.90 (±0.09)	2.81 (±0.07)

Diets were analyzed in triplicates. The values presented are the mean (±standard deviation).

**Table 3:** Primers used to amplify target genes.

	Primer	Nucleotide Sequence (5′-3′)	Amplicon Size (Base Pairs)
***PepT1***	Forward	AAGCTGGGACCGAGAAGATT	120
	Reverse	CGGCCAATAAAGTGGTTTCA	
***eEF1a1***	Forward	GTTGCTGCTGGTGTTGGTGAG	156
	Reverse	GAAACGCTTCTGGCTGTAAGG	
***α-tubulin***	Forward	GACTCGACCACAAGTTTGACC	142
	Reverse	GTTCCCACCTCTTCGTAATCC	
***β-actin***	Forward	GTATTGTCATGGACTCTGGTG	182
	Reverse	ACGTACGATTTCACGCTCAGC	

**Table 4:** Diet effect on growth and survival.

Group	Avg. Weight (mg)	Avg. Total Length (mm)	Survival (%)
LF-Intact	79.50 ^c^ (±30.00)	19.93 ^d^ (±2.49)	46.29 ^c^ (±9.20)
LF-Hydro	70.67 ^bc^ (±23.71)	18.60 ^c^ (±1.95)	33.78 ^b^ (±6.72)
Intact	51.67 ^ac^ (±14.74)	16.55 ^b^ (±2.25)	23.94 ^b^ (±8.69)
Hydro-A	44.33 ^ab^ (±4.04)	16.35 ^ab^ (±2.38)	11.69 ^a^ (±2.11)
Hydro-BCD	35.83 ^a^ (±6.37)	15.78 ^ab^ (±2.05)	11.33 ^a^ (±4.89)
Hydro-DCB	41.00 ^a^ (±7.81)	15.31 ^a^ (±2.88)	9.96 ^a^ (±2.85)
Artemia	72.32 ^bc^ (±10.98)	17.74 ^c^ (±1.55)	86.89 ^d^ (±1.81)

Values are presented as means (±S.D.). Letters indicate statistical significance between groups. The significance was determined using a One-Way ANOVA and a Tukey test with a *p* value < 0.05.

**Table 5:** Postprandial FAA composition in muscle.

Amino Acid (μM/g)	LF-Intact	LF-Hydro	Intact	Hydro-A	Hydro-BCD	Hydro-DCB	Artemia
**Aspartic Acid**	0.64 ^ab^ (±0.02)	0.65 ^ab^ (±0.01)	0.61 ^ab^ (±0.06)	0.61 ^ab^ (±0.05)	0.63 ^ab^ (±0.01)	0.70 ^b^ (±0.05)	0.53 ^a^ (±0.08)
**Glutamic Acid**	1.56 ^bc^ (±0.08)	1.63 ^c^ (±0.05)	1.41 ^ac^ (±0.05)	1.14 ^a^ (±0.11)	1.40 ^ac^ (±0.07)	1.35 ^ac^ (±0.03)	1.25 ^ab^ (±0.29)
**Asparagine**	0.38 ^a^ (±0.04)	0.50 ^ab^ (±0.02)	0.54 ^ab^ (±0.08)	0.46 ^ab^ (±0.09)	0.53 ^ab^ (±0.04)	0.57 ^b^ (±0.08)	0.45 ^ab^ (±0.05)
**Serine**	0.55 ^a^ (±0.11)	0.74 ^ab^ (±0.10)	0.58 ^a^ (±0.13)	0.60 ^a^ (±0.12)	0.88 ^b^ (±0.07)	0.93 ^b^ (±0.04)	0.55 ^a^ (±0.07)
**Glutamine**	0.63 ^ab^ (±0.04)	0.72 ^ab^ (±0.03)	0.79 ^b^ (±0.05)	0.53 ^a^ (±0.09)	0.71 ^ab^ (±0.06)	0.76 ^ab^ (±0.17)	0.64 ^ab^ (±0.05)
**Histidine**	0.73 ^b^ (±0.12)	0.69 ^b^ (±0.05)	0.61 ^b^ (±0.14)	0.61 ^b^ (±0.01)	0.56 ^b^ (±0.04)	0.60 ^b^ (±0.01)	0.28 ^a^ (±0.11)
**Glycine**	1.91 ^bc^ (±0.36)	2.55 ^c^ (±0.15)	1.35 ^b^ (±0.44)	1.31 ^ab^ (±0.22)	1.69 ^b^ (±0.30)	1.75 ^b^ (±0.03)	0.61 ^a^ (±0.04)
**Threonine**	0.70 ^b^ (±0.11)	0.94 ^c^ (±0.03)	0.61 ^b^ (±0.09)	0.58 ^ab^ (±0.09)	0.73 ^b^ (±0.06)	0.68 ^b^ (±0.02)	0.41 ^a^ (±0.08)
**Arginine**	0.09 ^b^ (±0.01)	0.12 ^c^ (±0.01)	0.10 ^b^ (±0.00)	0.09 ^b^ (±0.01)	0.11 ^bc^ (±0.01)	0.10 ^b^ (±0.01)	0.04 ^a^ (±0.01)
**Alanine**	0.83 ^abc^ (±0.19)	1.06 ^bd^ (±0.10)	0.82 ^ab^ (±0.23)	1.00 ^bc^ (±0.13)	1.22 ^cd^ (±0.03)	1.41 ^d^ (±0.02)	0.54 ^a^ (±0.13)
**Tyrosine**	0.20 ^bc^ (±0.01)	0.25 ^d^ (±0.01)	0.19 ^bc^ (±0.02)	0.17 ^b^ (±0.03)	0.22 ^cd^ (±0.01)	0.19 ^bc^ (±0.01)	0.09 ^a^ (±0.02)
**Lysine**	1.11 ^b^ (±0.09)	1.24 ^b^ (±0.03)	1.21 ^b^ (±0.07)	0.98 ^ab^ (±0.06)	1.16 ^b^ (±0.04)	1.12 ^b^ (±0.07)	0.73 ^a^ (±0.19)
**Methionine**	1.57 ^a^ (±0.06)	1.87 ^ab^ (±0.18)	1.74 ^ab^ (±0.18)	2.21 ^b^ (±0.20)	1.87 ^ab^ (±0.08)	1.95 ^ab^ (±0.11)	2.06 ^ab^ (±0.43)
**Valine**	1.95 (±0.07)	2.32 (±0.15)	2.01 (±0.05)	2.34 (±0.52)	1.99 (±0.09)	2.29 (±0.10)	2.41 (±0.13)
**Cysteine**	0.00	0.00	0.00	0.00	0.00	0.00	0.00
**Tryptophan**	0.12 ^ab^ (±0.03)	0.11 ^a^ (±0.01)	0.12 ^ab^ (±0.00)	0.08 ^a^ (±0.01)	0.16 ^b^ (±0.03)	0.12 ^ab^ (±0.00)	0.10 ^a^ (±0.01)
**Phenylalanine**	0.28 ^b^ (±0.02)	0.30 ^b^ (±0.02)	0.33 ^bc^ (±0.02)	0.28 ^b^ (±0.01)	0.39 ^c^ (±0.04)	0.33 ^b^ (±0.01)	0.19 ^a^ (±0.02)
**Isoleucine**	0.24 (±0.17)	0.15 (±0.03)	0.34 (±0.22)	0.11 (±0.03)	0.19 (±0.04)	0.14 (±0.03)	0.16 (±0.02)
**Leucine**	0.24 (±0.14)	0.38 (±0.05)	0.33 (±0.21)	0.35 (±0.05)	0.44 (±0.05)	0.40 (±0.04)	0.25 (±0.04)
**Proline**	0.35 ^a^ (±0.02)	0.47 ^ab^ (±0.07)	0.49 ^ab^ (±0.17)	0.87 ^cd^ (±0.03)	0.71 ^bc^ (±0.08)	1.07 ^d^ (±0.04)	0.58 ^ab^ (±0.12)
**IDAA**	7.03 ^ab^ (±0.28)	8.11 ^c^ (±0.26)	7.40 ^ac^ (±0.36)	7.65 ^ac^ (±0.63)	7.60 ^ac^ (±0.29)	7.74 ^bc^ (±0.13)	6.62 ^a^ (±0.45)
**DAA**	7.06 ^ad^ (±0.67)	8.57 ^cd^ (±0.16)	6.78 ^ac^ (±1.10)	6.70 ^ab^ (±0.84)	7.99 ^bcd^ (±0.34)	8.73 ^d^ (±0.31)	5.24 ^a^ (±0.71)
**TFAA**	14.10 ^ab^ (±0.95)	16.68 ^c^ (±0.41)	14.18 ^ac^ (±1.33)	14.35 ^ac^ (±1.10)	15.59 ^bc^ (±0.60)	16.46 ^bc^ (±0.37)	11.87 ^a^ (±1.11)

The values presented are postprandial levels, 2 h after feeding. Values are presented as means (±S.D). Superscript letters indicate statistical significance between groups. The significance was determined using a One-way ANOVA and an LSD test with a *p* value < 0.05. Indispensable amino acids (IDAA) = Ile, Leu, Lys, Met, Phe, Thr, Trp, Val, Arg, and His. Dispensable amino acids (DAA) = Ala, Asp, Asn, Glu, Gln, Gly, Pro, Ser, and Tyr. TFAA = total free amino acids.

**Table 6:** Postprandial FAA composition in muscle.

Amino Acid (μM/g)	LF-Intact	LF-Hydro	Intact	Hydro-A	Hydro-BCD	Hydro-DCB	Artemia
**Aspartic Acid**	0.62 (±0.02)	0.70 (±0.06)	0.75 (±0.03)	0.73 (±0.02)	0.72 (±0.06)	0.66 (±0.01)	0.46 (±0.01)
**Glutamic Acid**	1.34 (±0.05)	1.29 (±0.09)	1.45 (±0.09)	1.25 (±0.03)	1.45 (±0.08)	1.19 (±0.06)	0.93 (±0.05)
**Asparagine**	0.45 (±0.03)	0.42 (±0.04)	0.57 (±0.04)	0.50 (±0.01)	0.51 (±0.06)	0.43 (±0.01)	0.59 (±0.09)
**Serine**	0.30 (±0.04)	0.29 (±0.01)	0.51 (±0.06)	0.52 (±0.05)	0.66 (±0.09)	0.42 (±0.02)	0.72 (±0.13)
**Glutamine**	0.55 (±0.03)	0.52 (±0.04)	0.83 (±0.04)	0.68 (±0.02)	0.86 (±0.09)	0.52 (±0.02)	0.70 (±0.07)
**Histidine**	0.73 (±0.07)	0.54 (±0.09)	0.56 (±0.07)	0.42 (±0.07)	0.41 (±0.03)	0.34 (±0.02)	0.25 (±0.08)
**Glycine**	1.89 (±0.45)	2.64 (±0.49)	1.60 (±0.26)	1.43 (±0.25)	1.53 (±0.23)	1.47 (±0.14)	0.70 (±0.13)
**Threonine**	0.64 (±0.06)	0.88 (±0.13)	0.62 (±0.04)	0.54 (±0.06)	0.59 (±0.04)	0.51 (±0.04)	0.31 (±0.04)
**Arginine**	0.07 (±0.01)	0.05 (±0.00)	0.07 (±0.01)	0.06 (±0.01)	0.05 (±0.00)	0.04 (±0.00)	0.03 (±0.00)
**Alanine**	0.50 (±0.06)	0.36 (±0.04)	0.50 (±0.04)	0.57 (±0.07)	0.51 (±0.11)	0.40 (±0.01)	0.84 (±0.17)
**Tyrosine**	0.13 (±0.02)	0.19 (±0.02)	0.16 (±0.01)	0.14 (±0.01)	0.18 (±0.00)	0.15 (±0.01)	0.04 (±0.00)
**Lysine**	0.71 (±0.06)	0.79 (±0.05)	0.70 (±0.04)	0.60 (±0.05)	0.53 (±0.02)	0.45 (±0.05)	0.24 (±0.02)
**Methionine**	0.34 (±0.04)	0.34 (±0.04)	0.39 (±0.03)	0.34 (±0.05)	0.33 (±0.07)	0.55 (±0.04)	0.40 (±0.01)
**Valine**	1.91 (±0.09)	1.85 (±0.06)	1.83 (±0.08)	1.95 (±0.08)	1.63 (±0.04)	2.17 (±0.12)	1.80 (±0.03)
**Cysteine**	0.00	0.00	0.00	0.00	0.00	0.00	0.00
**Tryptophan**	0.05 (±0.00)	0.06 (±0.00)	0.08 (±0.00)	0.08 (±0.00)	0.10 (±0.01)	0.08 (±0.00)	0.06 (±0.01)
**Phenylalanine**	0.18 (±0.01)	0.21 (±0.02)	0.23 (±0.03)	0.22 (±0.00)	0.23 (±0.01)	0.22 (±0.01)	0.12 (±0.00)
**Isoleucine**	0.10 (±0.00)	0.06 (±0.01)	0.10 (±0.01)	0.10 (±0.01)	0.05 (±0.02)	0.03 (±0.00)	0.04 (±0.00)
**Leucine**	0.21 (±0.01)	0.17 (±0.02)	0.24 (±0.01)	0.20 (±0.01)	0.14 (±0.01)	0.12 (±0.01)	0.09 (±0.00)
**Proline**	0.54 (±0.06)	0.74 (±0.13)	0.66 (±0.03)	1.05 (±0.25)	0.98 (±0.16)	1.08 (±0.10)	1.33 (±0.07)
**IDAA**	4.94 (±0.26)	4.96 (±0.24)	4.82 (±0.26)	4.51 (±0.10)	4.05 (±0.13)	4.51 (±0.02)	3.35 (±0.11)
**DAA**	6.30 (±0.54)	7.16 (±0.68)	7.04 (±0.28)	6.86 (±0.63)	7.39 (±0.31)	6.36 (±0.27)	6.32 (±0.66)
**TFAA**	11.24 (±0.80)	12.12 (±0.92)	11.86 (±0.53)	11.37 (±0.63)	11.44 (±0.41)	10.85 (±0.25)	9.66 (±0.77)

The values presented are postprandial levels, 24 h after feeding. Values are presented as means (±S.D). Indispensable amino acids (IDAA) = Ile, Leu, Lys, Met, Phe, Thr, Trp, Val, Arg, and His. Dispensable amino acids (DAA) = Ala, Asp, Asn, Glu, Gln, Gly, Pro, Ser, and Tyr. TFAA = total free amino acids.

## Data Availability

Data can be available upon direct request to the corresponding author.
